# Supplementary material for: Applying a cytochrome c oxidase I barcode for Leishmania species typing
Source: PLoS One. 2024 Dec 2;19(12):e0309277. doi: 10.1371/journal.pone.0309277 (PMC11611135; doi:10.1371/journal.pone.0309277)
Supplement: S1 Table — IOC-L is the prefix use by the Oswaldo Cruz Institute’s Leishmania Collection (CLIOC) as part of the deposit code applied to Leishmania strains. (DOCX) [file pone.0309277.s001.docx]

**S1 Table. DNA sequences obtained from GenBank^®^, of the National Center for Biotechnology Information (NCBI), and from the Oswaldo Cruz Foundation’s *Leishmania* Collection database, corresponding to all available *coI* sequences from *Leishmania* spp. strains, for the evaluation of the *coI* target.**

|  | **IOC-L** | **International Code** | **Accession Number** | **Species** | **Isolation year** | **Country** | **State** | **City** | **Latitude** | **Longitude** |
| --- | --- | --- | --- | --- | --- | --- | --- | --- | --- | --- |
| 1 | - | - | LR697134 | *L. (V.) braziliensis* | - | - | - | - | - | - |
| 2 | - | MHOM/PE/1990/LCA04 | BK010881 | *L. (V.) peruviana* | 1990 | Peru | Ayacucho | - | - | - |
| 3 | - | MHOM/PA/1994/PSC-1 | BK010875 | *L. (V.) panamensis* | - | - | - | - | - | - |
| 4 | - | - | BK010883 | *L. (V.) shawi* | - | - | - | - | - | - |
| 5 | - | MHOM/BR/1981/M6426 | BK010879 | *L. (V.) lainsoni* | 1981 | Brasil | Pará | Benevides | 48W14'41.0 | 1S21'41.0" |
| 6 | - | MHOM/TN/2011/ZK47 | MT762287 | *L. (L.) infantum* | 2011 | Tunis | Kairouan | - | - | - |
| 7 | - | - | CP022652 | *L. (L.) donovani* | 1962 | Sudan | - | - |  |  |
| 8 | - | MHOM/SD/62/1S-Cl2D | FJ416603 | *L. (L.) donovani* | 1962 | Sudan | - | - | - | - |
| 9 | - | MHOM/CO/1993/UA-946 | MK570510 | *L. (V.) panamensis* | 1993 | Colombia | - | - | - | - |
| 10 | - | - | BK010874 | *Endotrypanum schaudinni* | 1980 | Brasil | Pará | - | - | - |
| 11 | - | - | LR697136 | *L. (S.) adleri* | - | - | - | - | - | - |
| 12 | 51 | MHOM/BR/1976/JOF | OR717017 | *L. (L.) major* | 1976 | Brazil | Minas Gerais | Caratinga | 19S47'23.0" | 42W08'21.0" |
| 13 | 59 | MHOM/BR/1973/M2269 | OR716953 | *L. (L.) amazonensis* | 1973 | Brazil | Pará | Magalhães Barata | 0S45'00.0" | 47W40'00.0" |
| 14 | 71 | MHOM/BR/1975/JOSEFA | OR716954 | *L. (L.) amazonensis* | 1975 | Brazil | Bahia | Ubaíra | 13S16'06.0" | 39W39'46.0" |
| 15 | 117 | MHOM/BZ/1958/LM5 | OR716925 | *L. (L.) mexicana* | 1958 | Belize | Belize | - | 17N10'60.0" | 88W28'60.0" |
| 16 | 133 | MHOM/IL/1977/LRC-L223 | OR716974 | *L. (L.) major* | 1977 | Israel | - | - | 31N27'59.0" | 35E23'17.0" |
| 17 | 183 | MCAN/BR/1973/LD70 | OR716975 | *L. (L.) major* | 1973 | Brazil | Minas Gerais | Conselheiro Pena | 19S10'20.0" | 41W28'20.0" |
| 18 | 185 | MHOM/BR/1977/LTB0016 | OR717014 | *L. (L.) amazonensis* | 1977 | Brazil | Bahia | Ubaíra | 13S16'06.0" | 39W39'46.0" |
| 19 | 186 | MHOM/VE/XXXX/L20 | OR716977 | *L. (L.) major* | - | Venezuela | - | - | - | - |
| 20 | 240 | MHOM/HN/1979/INC-4 | OR716901 | *L. (V.) panamensis* | 1979 | Honduras | El Paraíso | Danli | - | - |
| 21 | 243 | MHOM/CR/1983/SIA-034 | OR716906 | *L. (V.) panamensis* | 1983 | Costa Rica | San José | Acosta | 9N47'48.0" | 84W09'37.0" |
| 22 | 262 | MHOM/BR/1983/DAY | OR717015 | *L. (L.) amazonensis* | 1983 | Brazil | Maranhão | Buriticupu | 4S20'45.0" | 46W24'04.0" |
| 23 | 324 | MHOM/BR/1984/GJB | OR716970 | *L. (L.) amazonensis* | 1984 | Brazil | Goiás | Goiás | 15S55'60.0" | 50W07'60.0" |
| 24 | 536 | MHOM/BR/1985/CANDIDA | OR716971 | *L. (L.) amazonensis* | 1985 | Brazil | Bahia | Itaparica | 12S53'18.0" | 38W40'43.0" |
| 25 | 561 | MHOM/BZ/1982/BEL21 | OR716927 | *L. (L.) mexicana* | 1982 | Belize | Belize | - | 17N10'60.0" | 88W28'60.0" |
| 26 | 563 | MHOM/ET/1967/HU3 | OR716987 | *L. (L.) donovani* | 1967 | Ethiopia | Amara | Begemender | 37E00'00.0" | 12N30'00.0" |
| 27 | 565 | MHOM/BR/1975/M4147 | OR716918 | *L. (V.) guyanensis* | 1975 | Brazil | Pará | Almeirim | 0S52'04.0" | 52W32'06.0" |
| 28 | 568 | MHOM/VE/1974/PM-H3 | OR716951 | *L. (L.) venezuelensis* | 1974 | Venezuela | Lara | Barquisimeto | - | - |
| 29 | 571 | MHOM/AZ/1958/STRAINOD | OR716998 | *L. (L.) tropica* | 1958 | Azerbaijan | Gəncə | - | 40N40'58.0" | 46E21'38.0" |
| 30 | 572 | MHOM/AZ/1974/K27 | OR716999 | *L. (L.) tropica* | 1974 | Azerbaijan | Bakı | - | 40N23'13.0" | 49E53'27.0" |
| 31 | 574 | MRHO/UZ/1959/P-STRAIN | OR716978 | *L. (L.) major* | 1959 | Uzbekistan | Buxoro | - | 39N46'00.0" | 64E25'60.0" |
| 32 | 575 | IFLA/BR/1967/PH8 | OR716941 | *L. (L.) amazonensis* | 1967 | Brazil | Pará | Belém | 1S27'21.0" | 48W30'16.0" |
| 33 | 577 | MNYC/BZ/1962/M379 | OR716928 | *L. (L.) mexicana* | 1962 | Belize | Cayo | - | 17N05'54.0" | 88W56'29.0" |
| 34 | 579 | MHOM/BR/1974/PP75 | OR716995 | *L. (L.) infantum* | 1974 | Brazil | Bahia | Ituaçu | 13S48'48.0" | 41W17'48.0" |
| 35 | 580 | MHOM/ET/1971/L100 | OR682567 | *L. (L.) aethiopica* | 1971 | Ethiopia | - | - | - | - |
| 36 | 581 | MHOM/TM/1973/5-ASKH | OR716979 | *L. (L.) major* | 1973 | Turkmenistan | Ashkhabad | - | 37N55'60.0" | 58E22'00.0" |
| 37 | 584 | MHOM/VE/1976/JAP78 | OR716955 | *L. (L.) garnhami* | 1976 | Venezuela | - | - | - | - |
| 38 | 589 | MHOM/PE/1984/LH13 | OR716966 | *L. (L.) amazonensis* | 1984 | Peru | Pasco | - | - | - |
| 39 | 621 | MHOM/BR/1985/CLN | OR717016 | *L. (L.) amazonensis* | 1985 | Brazil | Bahia | - | - | - |
| 40 | 656 | MHOM/HN/1985/H-35 | OR716996 | *L. (L.) infantum* | 1985 | Honduras | Olancho | - | 15N11'60.0" | 86W14'31.0" |
| 41 | 704 | MHOM/BR/1986/LCD-MA | OR716963 | *L. (L.) amazonensis* | 1986 | Brazil | Maranhão | - | - | - |
| 42 | 719 | MHOM/CO/1984/CL-014 | OR716956 | *L. (L.) amazonensis* | 1984 | Colombia | Meta | - | - | - |
| 43 | 728 | MHOM/BR/1986/JGR | OR716957 | *L. (L.) amazonensis* | 1986 | Brazil | Bahia | Casa Nova | 8S55'48.0" | 41W26'13.0" |
| 44 | 729 | MHOM/BR/1986/RMSF | OR716958 | *L. (L.) amazonensis* | 1986 | Brazil | Bahia | Casa Nova | 8S55'48.0" | 41W26'13.0" |
| 45 | 778 | MHOM/BR/1986/BA-106 | OR716959 | *L. (L.) amazonensis* | 1986 | Brazil | Bahia | Ilhéus | 14S47'20.0" | 39W02'58.0" |
| 46 | 854 | ISQU/BR/1985/IM2264 | OR716921 | *L. (V.) naiffi* | 1985 | Brazil | Pará | - | - | - |
| 47 | 867 | MHOM/VE/1960/LTROD | OR716980 | *L. (L.) major* | 1960 | Venezuela | - | - | - | - |
| 48 | 876 | MHOM/BR/1987/BA-125 | OR716964 | *L. (L.) amazonensis* | 1987 | Brazil | Bahia | Alagoinhas | 12S08'08.0" | 38W25'09.0" |
| 49 | 888 | MCHO/EC/1982/LSP1 | OR717009 | *Leishmania equatorensis* | 1982 | Ecuador | Guayas | - | - | - |
| 50 | 889 | MSCI/EC/1982/LSP2 | OR717010 | *Leishmania equatorensis* | 1982 | Ecuador | Guayas | - | - | - |
| 51 | 895 | MHOM/EC/1987/G-09 | OR716981 | *L. (L.) major* | 1987 | Ecuador | Esmeraldas | Quininde | 0N19'60.0" | 79W28'60.0" |
| 52 | 897 | MDID/BR/1987/IM3217 | OR716965 | *L. (L.) amazonensis* | 1987 | Brazil | Amazonas | Presidente Figueiredo | 1S53'57.0" | 59W28'42.0" |
| 53 | 930 | MHOM/BR/1987/BA-89 | OR716940 | *L. (L.) amazonensis* | 1987 | Brazil | Bahia | - | - | - |
| 54 | 951 | MHOM/VE/1985/PM-H121 | OR716952 | *L. (L.) venezuelensis* | 1985 | Venezuela | Lara | Barquisimeto | 10N04'26.0" | 69W19'22.0" |
| 55 | 952 | MHOM/VE/1985/PM-H123 | OR716933 | *L. (L.) venezuelensis* | 1985 | Venezuela | Lara | Distrito Palavecino | 10N02'22.0" | 69W11'44.0" |
| 56 | 993 | MDAS/BR/1987/IM3281 | OR716920 | *L. (V.) naiffi* | 1987 | Brazil | Pará | São Félix do Xingu | 6S38'41.0" | 51W59'42.0" |
| 57 | 1009 | MHOM/BR/1988/SSC | OR716877 | *L. (V.) braziliensis* | 1988 | Brazil | Rio de Janeiro | Rio de Janeiro | 22S54'11.0" | 43W12'27.0" |
| 58 | 1015 | MHOM/MX/XXXX/MXPT5 | OR716923 | *L. (L.) mexicana* | - | Mexico | - | - | - | - |
| 59 | 1020 | MHOM/MX/XXXX/MXPT10 | OR716924 | *L. (L.) mexicana* | - | Mexico | - | - | - | - |
| 60 | 1023 | MHOM/BR/1981/M6426 | OR716922 | *L. (V.) lainsoni* | 1981 | Brazil | Pará | Benevides | 1S21'41.0" | 48W14'41.0" |
| 61 | 1043 | MHOM/PY/1987/FB | OR716935 | *L. (L.) amazonensis* | 1987 | Paraguay | Canindé | Villa Ygatim¡ | 24S04'48.0" | 55W30'00.0" |
| 62 | 1044 | MHOM/PY/1987/BN | OR716972 | *L. (L.) major* | 1987 | Paraguay | Canindé | Villa Ygatim¡ | 24S04'48.0" | 55W30'00.0" |
| 63 | 1060 | MHOM/BR/1983/IM1765 | OR716936 | *L. (L.) amazonensis* | 1983 | Brazil | Pará | Tucuruí | 3S45'58.0" | 49W40'21.0" |
| 64 | 1066 | IWHI/BR/1985/IM2322 | OR716913 | *L. (V.) shawi* | 1985 | Brazil | Pará | Breu Branco | 4S04'04.0" | 49W38'13.0" |
| 65 | 1067 | IWHI/BR/1985/IM2324 | OR682507 | *L. (V.) shawi* | 1985 | Brazil | Pará | - | - | - |
| 66 | 1068 | IWHI/BR/1985/IM2326 | OR716914 | *L. (V.) shawi* | 1985 | Brazil | Pará | - | - | - |
| 67 | 1071 | IFLA/BR/1985/IM2511 | OR716937 | *L. (L.) amazonensis* | 1985 | Brazil | Amazonas | - | - | - |
| 68 | 1134 | MHOM/VE/1987/PM-H149 | OR716929 | *L. (L.) venezuelensis* | 1987 | Venezuela | Lara | Union Eldorado | - | - |
| 69 | 1227 | MHOM/EC/1988/PAUTE-25 | OR716926 | *L. (L.) mexicana* | 1988 | Ecuador | Azuay | Paute | 2S46'59.0" | 78W43'59.0" |
| 70 | 1231 | MHOM/EC/1988/PAUTE-115 | OR716973 | *L. (L.) major* | 1988 | Ecuador | Azuay | Paute | 2S46'59.0" | 78W43'59.0" |
| 71 | 1245 | IGOM/PA/1985/E582.34 | OR682568 | *Leishmania colombiensis* | 1985 | Panama | Colón | - | 9N19'60.0" | 79W54'00.0" |
| 72 | 1246 | IPAN/PA/1985/E692.26 | OR717003 | *Leishmania colombiensis* | 1985 | Panama | Colón | - | 9N19'60.0" | 79W54'00.0" |
| 73 | 1247 | IGOM/PA/1985/E582.36 | OR717004 | *Leishmania colombiensis* | 1985 | Panama | Colón | - | 9N19'60.0" | 79W54'00.0" |
| 74 | 1252 | MHOM/NI/1988/XD45 | OR682506 | *L. (V.) panamensis* | 1988 | Nicaragua | Chontales | Los Chiles | 11N14'16.0" | 84W34'02.0" |
| 75 | 1266 | MCUN/BR/1983/IM1721 | OR682526 | *L. (V.) lainsoni* | 1983 | Brazil | Pará | Novo Repartimento | 3S45'58.0" | 49W40'21.0" |
| 76 | 1365 | MDAS/BR/1979/M5533 | OR682524 | *L. (V.) naiffi* | 1979 | Brazil | Pará | Almeirim | 1S31'24.0" | 52W34'54.0" |
| 77 | 1530 | MCOE/BR/1989/M5088 | OR717011 | *L. (P.) deanei* | 1989 | Brazil | Pará | - | - | - |
| 78 | 1545 | MCEB/BR/1984/M8408 | OR716915 | *L. (V.) shawi* | 1984 | Brazil | Pará | Parauapebas | 6S04'04.0" | 49W54'07.0" |
| 79 | 1730 | IYUI/BR/1992/IM255 | OR716938 | *L. (L.) amazonensis* | 1992 | Brazil | Rondônia | Porto Velho | 8S45'43.0" | 63W54'14.0" |
| 80 | 1734 | MHOM/BR/1991/IM3713 | OR716878 | *L. (V.) braziliensis* | 1991 | Brazil | Amazonas | Manaus | 4S05'07.0" | 63W08'29.0" |
| 81 | 1840 | MHOM/SU/1987/NARK | OR716976 | *L. (L.) major* | 1987 | Soviet Union | - | - | - | - |
| 82 | 1906 | MCAN/BR/1990/C35 | OR716879 | *L. (V.) braziliensis* | 1990 | Brazil | Ceará | - | - | - |
| 83 | 1907 | MCAN/BR/1991/C51 | OR716880 | *L. (V.) braziliensis* | 1991 | Brazil | Ceará | - | - | - |
| 84 | 1943 | MHOM/BR/1994/IM3946 | OR716885 | *L. (V.) braziliensis* | 1994 | Brazil | Amazonas | São Gabriel da Cachoeira | 0S07'49.0" | 67W05'21.0" |
| 85 | 2024 | MHOM/BR/1993/IM3939 | OR716939 | *L. (L.) amazonensis* | 1993 | Brazil | Pará | Alenquer | 1S56'31.0" | 54W44'17.0" |
| 86 | 2064 | MRHO/CN/1988/KXG-2 | OR716984 | *L. (L.) turanica* | 1988 | China | - | - | - | - |
| 87 | 2073 | MHOM/BR/1995/VLNC | OR716886 | *L. (V.) braziliensis* | 1995 | Brazil | Rio de Janeiro | Parati | 23S13'05.0" | 44W42'47.0" |
| 88 | 2122 | MHOM/BR/1995/HV-7 | OR716887 | *L. (V.) braziliensis* | 1995 | Brazil | Espírito Santo | Viana | 20S23'22.0" | 40W29'36.0" |
| 89 | 2125 | MHOM/BR/1995/HAC-6 | OR716888 | *L. (V.) braziliensis* | 1995 | Brazil | Espírito Santo | Afonso Cláudio | 20S04'27.0" | 41W07'26.0" |
| 90 | 2160 | MHOM/BR/1996/SBS | OR716889 | *L. (V.) braziliensis* | 1996 | Brazil | Bahia | Jequi‚ | 13S51'27.0" | 40W05'01.0" |
| 91 | 2266 | MHOM/VE/XXXX/PM-H241 | OR717005 | *Leishmania colombiensis* | - | Venezuela | Lara | - | - | - |
| 92 | 2267 | MHOM/VE/1996/PM-H242 | OR717006 | *Leishmania colombiensis* | 1996 | Venezuela | Lara | Urdaneta | 10N39'09.0" | 69W42'20.0" |
| 93 | 2268 | MHOM/VE/XXXX/PM-H243 | OR717007 | *Leishmania colombiensis* | - | Venezuela | Lara | - | - | - |
| 94 | 2269 | MHOM/VE/XXXX/PM-H244 | OR717008 | *Leishmania colombiensis* | - | Venezuela | Lara | Quibor | 9N55'41.0" | 69W34'40.0" |
| 95 | 2272 | MHOM/ET/1967/L82;HV3;LV9 | OR716985 | *L. (L.) donovani* | 1967 | Ethiopia | - | - | - | - |
| 96 | 2274 | RLIZ/SD/XXXX/LV31 | OR717001 | *L. (S.) hoogstraali* | 1963 | Sudan | - | - | - | - |
| 97 | 2276 | MCOE/PA/1965/C-8 | OR716985 | *L. (P.) hertigi* | 1965 | Panama | - | - | - | - |
| 98 | 2277 | MCOE/PA/1972/C-119 | OR717013 | *L. (P.) hertigi* | 1972 | Panama | - | - | - | - |
| 99 | 2278 | RTAR/SN/1967/LV108 | OR717002 | *L. (S.) tarentolae* | 1967 | Senegal | - | - | - | - |
| 100 | 2301 | MHOM/BO/1997/LP-0017 | OR716988 | *L. (L.) infantum* | 1997 | Bolivia | La Paz | Tai Piplaya | 15S48'00.0" | 67W10'60.0" |
| 101 | 2307 | MHOM/BO/1996/CJ-13 | OR716934 | *L. (L.) amazonensis* | 1996 | Bolivia | La Paz | Cajuata | 16S49'12.0" | 67W15'00.0" |
| 102 | 2309 | MHOM/BO/1996/CJ-34 | OR716930 | *L. (L.) amazonensis* | 1996 | Bolivia | La Paz | Cajuata | 16S49'12.0" | 67W15'00.0" |
| 103 | 2325 | INUN/BO/1996/CJC | OR716931 | *L. (L.) amazonensis* | 1996 | Bolivia | La Paz | Cajuata | 16S49'12.0" | 67W15'00.0" |
| 104 | 2332 | XXXX/BO/1998/AA-101 | OR716932 | *L. (L.) amazonensis* | 1998 | Bolivia | La Paz | Chulumani | 16S24'32.0" | 67W31'40.0" |
| 105 | 2334 | MHOM/BR/1997/NMT-MAO 202P | OR682508 | *L. (V.) guyanensis* | 1997 | Brazil | Amazonas | Manaus | 3S06'07.0" | 60W01'30.0" |
| 106 | 2335 | MHOM/BR/1997/NMT-MAO 203P | OR682509 | *L. (V.) guyanensis* | 1997 | Brazil | Amazonas | Manaus | 3S06'07.0" | 60W01'30.0" |
| 107 | 2336 | MHOM/BR/1997/NMT-MAO 203G | OR682510 | *L. (V.) guyanensis* | 1997 | Brazil | Amazonas | Manaus | 3S06'07.0" | 60W01'30.0" |
| 108 | 2337 | MHOM/BR/1997/NMT-MAO 210P | OR682511 | *L. (V.) guyanensis* | 1997 | Brazil | Amazonas | Manaus | 3S06'07.0" | 60W01'30.0" |
| 109 | 2338 | MHOM/BR/1997/NMT-MAO 212P | OR682522 | *L. (V.) guyanensis* | 1997 | Brazil | Amazonas | Manaus | 3S06'07.0" | 60W01'30.0" |
| 110 | 2341 | MHOM/BR/1997/NMT-MAO 223P | OR682512 | *L. (V.) guyanensis* | 1997 | Brazil | Amazonas | Manaus | 3S06'07.0" | 60W01'30.0" |
| 111 | 2356 | MHOM/BR/1997/NMT-MAO 246P | OR682513 | *L. (V.) guyanensis* | 1997 | Brazil | Amazonas | - | - | - |
| 112 | 2371 | MHOM/BR/1997/NMT-MAO 292P | OR682523 | *L. (V.) guyanensis* | 1997 | Brazil | Amazonas | Manaus | 3S06'07.0" | 60W01'30.0" |
| 113 | 2372 | MHOM/BR/1997/NMT-MAO 292G | OR682514 | *L. (V.) guyanensis* | 1997 | Brazil | Amazonas | Manaus | 3S06'07.0" | 60W01'30.0" |
| 114 | 2389 | MHOM/BR/1997/NMT-MAO 307P | OR682515 | *L. (V.) guyanensis* | 1997 | Brazil | Amazonas | Manaus | 3S06'07.0" | 60W01'30.0" |
| 115 | 2396 | MHOM/BR/1997/NMT-MAO 315P | OR682516 | *L. (V.) guyanensis* | 1997 | Brazil | Amazonas | Manaus | 3S06'07.0" | 60W01'30.0" |
| 116 | 2398 | MHOM/BR/1997/NMT-MAO 317P | OR682517 | *L. (V.) guyanensis* | 1997 | Brazil | Amazonas | Manaus | 3S06'07.0" | 60W01'30.0" |
| 117 | 2405 | MHOM/BR/1997/NMT-MAO 325P | OR682518 | *L. (V.) guyanensis* | 1997 | Brazil | Amazonas | - | - | - |
| 118 | 2410 | MHOM/BR/1997/NMT-MAO 315P | OR682519 | *L. (V.) guyanensis* | 1997 | Brazil | Amazonas | - | - | - |
| 119 | 2459 | MHOM/BR/2000/JLS | OR716942 | *L. (L.) amazonensis* | 2000 | Brazil | Rio de Janeiro | - | - | - |
| 120 | 2463 | MHOM/BR/2001/JOLIVAL | OR716895 | *L. (V.) braziliensis* | 2001 | Brazil | Bahia | Presidente Tancredo Neves | 13S26'51.0" | 39W25'14.0" |
| 121 | 2468 | MHOM/BR/2001/LTCP14183 | OR716896 | *L. (V.) braziliensis* | 2001 | Brazil | Bahia | Presidente Tancredo Neves | 13S26'51.0" | 39W25'14.0" |
| 122 | 2475 | MHOM/BR/2001/LTCP14214 | OR716897 | *L. (V.) braziliensis* | 2001 | Brazil | Bahia | - | - | - |
| 123 | 2476 | MHOM/BR/2001/LTCP14278 | OR716898 | *L. (V.) braziliensis* | 2001 | Brazil | Bahia | - | - | - |
| 124 | 2479 | MHOM/BR/1999/LTCP13088 | OR716944 | *L. (L.) amazonensis* | 1999 | Brazil | Bahia | Presidente Tancredo Neves | 13S26'51.0" | 39W25'14.0" |
| 125 | 2481 | MHOM/BR/2000/LTCP13490 | OR716883 | *L. (V.) braziliensis* | 2000 | Brazil | Bahia | Presidente Tancredo Neves | 13S26'51.0" | 39W25'14.0" |
| 126 | 2491 | MHOM/BR/2002/NMT-RBO 005 | OR716884 | *L. (V.) braziliensis* | 2002 | Brazil | Acre | Rio Branco | - | - |
| 127 | 2497 | MHOM/BR/2002/NMT-RBO 027P | OR682528 | *L. (V.) lainsoni* | 2002 | Brazil | Acre | Rio Branco | - | - |
| 128 | 2501 | MHOM/BR/2002/NMT-RBO037 | OR682493 | *L. (V.) braziliensis* | 2002 | Brazil | Acre | Rio Branco | - | - |
| 129 | 2515 | MHOM/BR/2001/TSS | OR716899 | *L. (V.) braziliensis* | 2000 | Brazil | Pernambuco | Amaraji | 8S22'38.0" | 35W27'01.0" |
| 130 | 2571 | MHOM/BR/2003/NJS | OR716943 | *L. (L.) amazonensis* | 2003 | Brazil | Mato Grosso do Sul | Bonito | 21S07'16.0" | 56W28'55.0" |
| 131 | 2586 | MHOM/BR/2003/ERSS | OR716967 | *L. (L.) amazonensis* | 2003 | Brazil | Mato Grosso do Sul | Campo Grande | 20S26'34.0" | 54W38'47.0" |
| 132 | 2674 | MHOM/BR/2002/GR | OR716968 | *L. (L.) amazonensis* | 2002 | Brazil | Mato Grosso do Sul | Bela Vista | 22S06'32.0" | 56W31'16.0" |
| 133 | 2679 | MHOM/BR/2002/RSRR | OR716969 | *L. (L.) amazonensis* | 2002 | Brazil | Mato Grosso do Sul | Bela Vista | 22S06'32.0" | 56W31'16.0" |
| 134 | 2685 | MHOM/BR/2002/BMB | OR682500 | *L. (V.) braziliensis* | 2002 | Brazil | Mato Grosso do Sul | Bela Vista | 22S06'32.0" | 56W31'16.0" |
| 135 | 2689 | ITUB/BR/1977/M4964 | OR682503 | *L. (V.) utingensis* | 1977 | Brazil | Pará | Belém | 1S25'01.0" | 48W24'37.0" |
| 136 | 2690 | MHOM/BR/1966/M15733 | OR682501 | *L. (V.) lindenbergi* | 1966 | Brazil | Pará | Belém | 1S27'21.0" | 48W30'16.0" |
| 137 | 2692 | MHOM/BR/1995/ANAMARIA | OR682520 | *L. (V.) guyanensis* | 1995 | Brazil | Maranhão | Pinheiro | 2S31'17.0" | 45W04'57.0" |
| 138 | 2728 | MHOM/TN/1988/TN 435 | OR716983 | *L. (L.) major* | 1988 | Tunisia | - | - | - | - |
| 139 | 2730 | MHOM/BR/2003/phufms-43 | OR716960 | *L. (L.) amazonensis* | 2003 | Brazil | Mato Grosso do Sul | Campo Grande | 20S26'34.0" | 54W38'47.0" |
| 140 | 2735 | MHOM/BR/2003/phufms-25 | OR716945 | *L. (L.) amazonensis* | 2003 | Brazil | Mato Grosso do Sul | - | - | - |
| 141 | 2737 | MHOM/BR/2004/phufms-136 | OR716961 | *L. (L.) amazonensis* | 2004 | Brazil | Mato Grosso do Sul | Campo Grande | 20S26'34.0" | 54W38'47.0" |
| 142 | 2738 | MHOM/BR/2004/phufms-158 | OR716946 | *L. (L.) amazonensis* | 2004 | Brazil | Mato Grosso do Sul | Água Clara | 20S26'53.0" | 52W52'41.0" |
| 143 | 2740 | MHOM/BR/2003/phufms-90 | OR716962 | *L. (L.) amazonensis* | 2003 | Brazil | Mato Grosso do Sul | Campo Grande | 20S26'34.0" | 54W38'47.0" |
| 144 | 2798 | MHOM/SD/XXXX/MW1(S01) | OR716982 | *L. (L.) major* | - | Sudan | - | - | - | - |
| 145 | 2800 | MHOM/SD/2000/MW29(S03) | OR716986 | *L. (L.) donovani* | 2000 | Sudan | - | - | - | - |
| 146 | 2801 | MHOM/SD/2000/MW12(S04) | OR717000 | *L. (L.) archibaldi* | 2000 | Sudan | - | - | - | - |
| 147 | 2830 | MHOM/BR/1995/LTCP9986 | OR716947 | *L. (L.) amazonensis* | 1995 | Brazil | Bahia | Presidente Tancredo Neves | 13S26'51.0" | 39W25'14.0" |
| 148 | 2831 | MHOM/BR/1994/LTCP8137 | OR716948 | *L. (L.) amazonensis* | 1994 | Brazil | Bahia | Presidente Tancredo Neves | 13S26'51.0" | 39W25'14.0" |
| 149 | 2872 | MHOM/BR/2002/NMT-LTCP 14451-P | OR716900 | *L. (V.) braziliensis* | 2002 | Brazil | Bahia | Presidente Tancredo Neves | 13S26'51.0" | 39W25'14.0" |
| 150 | 2889 | MHOM/BR/2002/NMT-LTCP 14616-P | OR716890 | *L. (V.) braziliensis* | 2002 | Brazil | Bahia | Presidente Tancredo Neves | 13S26'51.0" | 39W25'14.0" |
| 151 | 2906 | MHOM/BR/2002/LPC-RPV | OR716989 | *L. (L.) infantum* | 2002 | Brazil | Minas Gerais | - | - | - |
| 152 | 2918 | MHOM/BR/2006/ICA | OR716891 | *L. (V.) braziliensis* | 2006 | Brazil | Rio de Janeiro | Teresópolis | 22S25'01.0" | 42W58'32.0" |
| 153 | 2927 | MHOM/BR/2001/NMT-LTCP14369-P | OR716892 | *L. (V.) braziliensis* | 2001 | Brazil | Bahia | Presidente Tancredo Neves | 13S26'51.0" | 39W25'14.0" |
| 154 | 2950 | MHOM/BR/2006/CEN | OR716881 | *L. (V.) braziliensis* | 2006 | Brazil | Pernambuco | Paudalho | 7S54'12.0" | 35W10'19.0" |
| 155 | 2951 | MHOM/BR/2006/CM | OR716882 | *L. (V.) braziliensis* | 2006 | Brazil | Pernambuco | Paudalho | 7S54'12.0" | 35W10'19.0" |
| 156 | 2963 | MHOM/BR/2007/033-MECM | OR682521 | *L. (V.) guyanensis* | 2007 | Brazil | Amazonas | Manaus | 3S06'07.0" | 60W01'30.0" |
| 157 | 2990 | MCAN/PY/2006/AS4 | OR716990 | *L. (L.) infantum* | 2006 | Paraguay | Central | - | 25S16'55.0" | 57W38'06.0" |
| 158 | 3007 | MHOM/BR/2003/IRCF | OR716919 | *L. (V.) naiffi* | 2003 | Brazil | Rio de Janeiro | Rio de Janeiro | 22S54'10.0" | 43W12'27.0" |
| 159 | 3013 | MHOM/BR/2007/ER | OR716917 | *L. (V.) guyanensis* | 2007 | Brazil | Amazonas | Rio Preto da Eva | 2S41'58.0" | 59W41'59.0" |
| 160 | 3034 | MHOM/PY/2007/AS-8 | OR716991 | *L. (L.) infantum* | 2007 | Paraguay | Asunción | - | 25S16'00.0" | 57W37'60.0" |
| 161 | 3049 | MCAN/BR/2007/LIBPI-60 | OR716992 | *L. (L.) infantum* | 2007 | Brazil | Piauí | Teresina | 5S05'21.0" | 42W48'07.0" |
| 162 | 3053 | MHOM/BR/2008/RJS | OR716993 | *L. (L.) infantum* | 2008 | Brazil | Pernambuco | Recife | 8S02'51.0" | 34W56'46.0" |
| 163 | 3068 | MCAN/BR/2008/CP-18 | OR716997 | *L. (L.) infantum* | 2008 | Brazil | Espírito Santo | Pancas | 19S13'31.0" | 40W51'05.0" |
| 164 | 3072 | MHOM/BR/2008/NC | OR716893 | *L. (V.) braziliensis* | 2008 | Brazil | Rio de Janeiro | Rio de Janeiro | 22S52'46.0" | 43W14'37.0" |
| 165 | 3090 | MHOM/BR/1998/LTCP-HR | OR716894 | *L. (V.) braziliensis* | 1998 | Brazil | Bahia | Salvador | 12S59'36.0" | 38W31'13.0" |
| 166 | 3132 | MCAN/BR/2009/PANTERA-MO I | OR682529 | *L. (L.) infantum* | 2009 | Brazil | Mato Grosso | Cuiabá | 15S34'39.0" | 56W01'58.0" |
| 167 | 3203 | MCAN/BR/2010/AKIRA II | OR682530 | *L. (L.) infantum* | 2010 | Brazil | Mato Grosso | Cuiabá | 15S35'46.0" | 56W05'48.0" |
| 168 | 3204 | MCAN/BR/2010/BOLINHA I | OR682531 | *L. (L.) infantum* | 2010 | Brazil | Mato Grosso | Cuiabá | 15S35'46.0" | 56W05'48.0" |
| 169 | 3206 | MCAN/BR/2010/LEAO II3 | OR682532 | *L. (L.) infantum* | 2010 | Brazil | Mato Grosso | Cuiabá | 15S35'46.0" | 56W05'48.0" |
| 170 | 3208 | MCAN/BR/2010/ZEUS | OR682533 | *L. (L.) infantum* | 2010 | Brazil | Mato Grosso | Cuiabá | 15S35'48.0" | 56W05'49.0" |
| 171 | 3219 | MCAN/BR/2010/TITÃ I | OR682534 | *L. (L.) infantum* | 2010 | Brazil | Mato Grosso | Cuiabá | 15S35'48.0" | 56W05'49.0" |
| 172 | 3226 | MCAN/BR/2010/CHITARA II | OR682535 | *L. (L.) infantum* | 2010 | Brazil | Mato Grosso | Cuiabá | 15S35'48.0" | 56W05'49.0" |
| 173 | 3233 | MCAN/BR/2010/DIANA III | OR682536 | *L. (L.) infantum* | 2010 | Brazil | Rio Grande do Sul | Uruguaiana | 29S45'17.0" | 57W05'18.0" |
| 174 | 3239 | MHOM/BR/2010/GFS | OR682504 | *L. (V.) braziliensis* | 2011 | Brazil | Pernambuco | Moreno | 8S07'07.0" | 35W05'32.0" |
| 175 | 3249 | MCAN/BR/2009/CLV3 | OR716994 | *L. (L.) infantum* | 2009 | Brazil | São Paulo | Embu das Artes | 23S38'56.0" | 46W51'07.0" |
| 176 | 3253 | MCAN/BR/2009/CLV14 | OR682537 | *L. (L.) infantum* | 2009 | Brazil | São Paulo | Embu das Artes | 23S38'56.0" | 46W51'07.0" |
| 177 | 3254 | MCAN/BR/2009/CLV17 | OR682538 | *L. (L.) infantum* | 2009 | Brazil | São Paulo | Embu das Artes | 23S38'56.0" | 46W51'07.0" |
| 178 | 3256 | MCAN/BR/2009/CLV22 | OR682539 | *L. (L.) infantum* | 2009 | Brazil | São Paulo | Embu das Artes | 23S38'56.0" | 46W51'07.0" |
| 179 | 3257 | MCAN/BR/2011/IMTS-14 | OR682540 | *L. (L.) infantum* | 2009 | Brazil | São Paulo | Embu das Artes | 23S38'56.0" | 46W51'07.0" |
| 180 | 3281 | MHOM/BR/2011/FdeAS-02 | OR716949 | *L. (L.) amazonensis* | 2011 | Brazil | Rondônia | Guajará-Mirim | 10S46'58.0" | 65W20'22.0" |
| 181 | 3284 | MHOM/CO/2009/6695 | OR716902 | *L. (V.) panamensis* | 2009 | Colombia | Chocó | Acandi | 8N30'51.0" | 77W16'53.0" |
| 182 | 3285 | MDID/CO/2009/95 | OR716903 | *L. (V.) panamensis* | 2009 | Colombia | Chocó | Acandi | 8N30'51.0" | 77W16'53.0" |
| 183 | 3286 | MDID/CO/2009/98 | OR716904 | *L. (V.) panamensis* | 2009 | Colombia | Chocó | Acandi | 8N30'51.0" | 77W16'53.0" |
| 184 | 3287 | MHOM/CO/2009/6547 | OR716905 | *L. (V.) panamensis* | 2009 | Colombia | Chocó | Acandi | 8N30'51.0" | 77W16'53.0" |
| 185 | 3288 | MHOM/CO/2009/6658 | OR716907 | *L. (V.) panamensis* | 2009 | Colombia | Chocó | Acandi | 8N30'51.0" | 77W16'53.0" |
| 186 | 3293 | MHOM/CO/2009/6638 | OR716908 | *L. (V.) panamensis* | 2009 | Colombia | Chocó | Acandi | 8N30'51.0" | 77W16'53.0" |
| 187 | 3294 | MHOM/CO/2009/6695 | OR716909 | *L. (V.) panamensis* | 2009 | Colombia | Chocó | Acandi | 8N30'51.0" | 77W16'53.0" |
| 188 | 3297 | MHOM/CO/2009/6596 | OR716910 | *L. (V.) panamensis* | 2009 | Colombia | Chocó | Acandi | 8N30'51.0" | 77W16'53.0" |
| 189 | 3301 | MHOM/CO/2009/6592 | OR716911 | *L. (V.) panamensis* | 2009 | Colombia | Chocó | Acandi | 8N30'51.0" | 77W16'53.0" |
| 190 | 3302 | MHOM/CO/2009/6633 | OR716912 | *L. (V.) panamensis* | 2009 | Colombia | Chocó | Acandi | 8N30'51.0" | 77W16'53.0" |
| 191 | 3310 | MHOM/BR/2011/S50 | OR682525 | *L. (V.) naiffi* | 2010 | Brazil | Pará | Prainha | 1S48'02.0" | 53W28'49.0" |
| 192 | 3311 | MHOM/BR/2011/S55 | OR716950 | *L. (L.) amazonensis* | 2010 | Brazil | Pará | Santarém | 2S26'37.0" | 54W42'31.0" |
| 193 | 3315 | MHOM/BR/2011/57-GBS | OR682527 | *L. (V.) lainsoni* | 2011 | Brazil | Pará | Santarém | 2S26'37.0" | 54W42'31.0" |
| 194 | 3328 | MHOM/BR/2011/COS | OR682541 | *L. (L.) infantum* | 2011 | Brazil | Pernambuco | Santa Cruz do Capibaribe | 7S57'27.0" | 36W12'17.0" |
| 195 | 3330 | MHOM/BR/2011/Diag 1367 | OR682542 | *L. (L.) infantum* | 2011 | Brazil | Rio Grande do Norte | Natal | 5S47'42.0" | 35W12'34.0" |
| 196 | 3331 | MHOM/BR/2011/Diag 1362 | OR682543 | *L. (L.) infantum* | 2011 | Brazil | Rio Grande do Norte | Natal | 5S47'42.0" | 35W12'34.0" |
| 197 | 3333 | MCAN/BR/2011/PV 127 | OR682544 | *L. (L.) infantum* | 2011 | Brazil | Rio Grande do Norte | Natal | 5S47'42.0" | 35W12'34.0" |
| 198 | 3335 | MCAN/BR/2011/PV 128 | OR682545 | *L. (L.) infantum* | 2011 | Brazil | Rio Grande do Norte | Natal | 5S47'42.0" | 35W12'34.0" |
| 199 | 3336 | MHOM/BR/2011/TC 03 | OR682546 | *L. (L.) infantum* | 2011 | Brazil | Rio Grande do Norte | Natal | 5S47'42.0" | 35W12'34.0" |
| 200 | 3337 | MHOM/BR/2011/TC 05 | OR682547 | *L. (L.) infantum* | 2011 | Brazil | Rio Grande do Norte | Natal | 5S47'42.0" | 35W12'34.0" |
| 201 | 3338 | MHOM/BR/2011/TC 14 | OR682548 | *L. (L.) infantum* | 2011 | Brazil | Rio Grande do Norte | Natal | 5S47'42.0" | 35W12'34.0" |
| 202 | 3339 | MHOM/BR/2011/TC 18 | OR682549 | *L. (L.) infantum* | 2011 | Brazil | Rio Grande do Norte | Natal | 5S47'42.0" | 35W12'34.0" |
| 203 | 3340 | MHOM/BR/2011/TC 28 | OR682550 | *L. (L.) infantum* | 2011 | Brazil | Rio Grande do Norte | Natal | 5S47'42.0" | 35W12'34.0" |
| 204 | 3341 | MHOM/BR/2011/TC 50 | OR682551 | *L. (L.) infantum* | 2011 | Brazil | Rio Grande do Norte | Natal | 5S47'42.0" | 35W12'34.0" |
| 205 | 3342 | MHOM/BR/2011/TC 65 | OR682552 | *L. (L.) infantum* | 2011 | Brazil | Rio Grande do Norte | Natal | 5S47'42.0" | 35W12'34.0" |
| 206 | 3343 | MHOM/BR/2011/TC 95 | OR682553 | *L. (L.) infantum* | 2011 | Brazil | Rio Grande do Norte | Natal | 5S47'42.0" | 35W12'34.0" |
| 207 | 3344 | MHOM/BR/2011/TC 96 | OR682554 | *L. (L.) infantum* | 2011 | Brazil | Rio Grande do Norte | Natal | 5S47'42.0" | 35W12'34.0" |
| 208 | 3346 | MHOM/BR/2011/TC 105 | OR682555 | *L. (L.) infantum* | 2011 | Brazil | Rio Grande do Norte | Natal | 5S47'42.0" | 35W12'34.0" |
| 209 | 3348 | MHOM/BR/2011/TC 118 | OR682556 | *L. (L.) infantum* | 2011 | Brazil | Rio Grande do Norte | Natal | 5S47'42.0" | 35W12'34.0" |
| 210 | 3368 | MHOM/BR/2011/AD1 | OR682557 | *L. (L.) infantum* | 2010 | Brazil | Minas Gerais | Belo Horizonte | 19S55'15.0" | 43W56'16.0" |
| 211 | 3369 | MHOM/BR/2011/AD2 | OR682558 | *L. (L.) infantum* | 2011 | Brazil | Minas Gerais | Belo Horizonte | 19S55'15.0" | 43W56'16.0" |
| 212 | 3376 | MCAN/BR/2012/Nina | OR682559 | *L. (L.) infantum* | 2012 | Brazil | São Paulo | São José do Rio Preto | 20S49'11.0" | 49W22'46.0" |
| 213 | 3377 | ARHI/BR/2011/2595(3) | OR682560 | *L. (L.) infantum* | 2011 | Brazil | Distrito Federal | - | - | - |
| 214 | 3378 | MCAN/BR/2010/CA1 | OR682561 | *L. (L.) infantum* | 2010 | Brazil | Minas Gerais | Belo Horizonte | 19S55'15.0" | 43W56'16.0" |
| 215 | 3379 | MCAN/BR/2010/CA2 | OR682562 | *L. (L.) infantum* | 2010 | Brazil | Minas Gerais | Belo Horizonte | 19S55'15.0" | 43W56'16.0" |
| 216 | 3381 | MCAN/BR/2010/CA4 | OR682563 | *L. (L.) infantum* | 2010 | Brazil | Minas Gerais | Belo Horizonte | 19S55'15.0" | 43W56'16.0" |
| 217 | 3384 | MHOM/BR/2012/AD6 | OR682564 | *L. (L.) infantum* | 2012 | Brazil | Minas Gerais | Belo Horizonte | 19S55'15.0" | 43W56'16.0" |
| 218 | 3385 | MHOM/BR/2012/AD7 | OR682565 | *L. (L.) infantum* | 2012 | Brazil | Minas Gerais | Belo Horizonte | 19S55'15.0" | 43W56'16.0" |
| 219 | 3388 | MHOM/BR/2011/TC 86 | OR682566 | *L. (L.) infantum* | 2011 | Brazil | Rio Grande do Norte | Natal | 5S47'42.0" | 35W12'34.0" |
| 220 | 3451 | MHOM/BR/2006/LSC128 | OR682505 | *L. (V.) braziliensis* | 2006 | Brazil | Santa Catarina | Florianópolis | 27S35'48.0" | 48W32'57.0" |
| 221 | 3481 | MHOM/BR/2013/18LTA-MLF | OR716916 | *L. (V.) shawi* | 2013 | Brazil | Amazonas | Manaus | 3S06'07.0" | 60W01'30.0" |
| 222 | 3658 | MHOM/PE/2002/LC2206 | OR682494 | *L. (V.) braziliensis* | 2002 | Peru | Ucayali | - | - | - |
| 223 | 3659 | MHOM/PE/XXXX/LC2940 | OR682495 | *L. (V.) peruviana* | - | Peru | - | - | - | - |
| 224 | 3660 | MHOM/PE/XXXX/LC2511 | OR682496 | *L. (V.) braziliensis* | - | Peru | - | - | - | - |
| 225 | 3662 | MHOM/PE/2003/LH2287 | OR682497 | *L. (V.) braziliensis* | 2003 | Peru | Cusco | - | - | - |
| 226 | 3663 | MHOM/PE/1991/LC1586 | OR682498 | *L. (V.) braziliensis* | 1991 | Peru | Cusco | Paucartambo | 13S18'50.0" | 71W35'35.0" |
| 227 | 3664 | MHOM/PE/2003/LH2868 | OR682502 | *L. (V.) peruviana* | 2003 | Peru | Cajamarca | - | - | - |
| 228 | 3665 | MHOM/PE/1991/LC1580 | OR682499 | *L. (V.) braziliensis* | 1991 | Peru | Cusco | Pilcopata | 13S03'21.0" | 71W32'39.0" |

IOC-L is the prefix use by the Oswaldo Cruz Institute’s Leishmania Collection (CLIOC) as part of the deposit code applied to *Leishmania* strains.
