# Supplementary material for: Applying a cytochrome c oxidase I barcode for Leishmania species typing
Source: PLoS One. 2024 Dec 2;19(12):e0309277. doi: 10.1371/journal.pone.0309277 (PMC11611135; doi:10.1371/journal.pone.0309277)
Supplement: S2 Table — (DOCX) [file pone.0309277.s002.docx]

**S2 Table. Between Groups Mean Distance (BGMD), a distance-based indicator, showing the average number of base differences of all sequence pairs, between the proposed species groups.**

| Group 1 | Group 2 | Distance | Standard Error |
| --- | --- | --- | --- |
| *L. (V.) braziliensis* | *L. (V.) peruviana* | 0.0043044752 | 0.0017845301 |
| *L. (V.) braziliensis* | *L. (V.) panamensis* | 0.0200300136 | 0.0058803216 |
| *L. (V.) peruviana* | *L. (V.) panamensis* | 0.0182744333 | 0.0058844741 |
| *L. (V.) braziliensis* | *L. (V.) shawi* | 0.0271024298 | 0.0070863977 |
| *L. (V.) peruviana* | *L. (V.) shawi* | 0.0240384842 | 0.0069203362 |
| *L. (V.) panamensis* | *L. (V.) shawi* | 0.0175921448 | 0.0058943813 |
| *L. (V.) braziliensis* | *L. (V.) guyanensis* | 0.0265781084 | 0.0070200058 |
| *L. (V.) peruviana* | *L. (V.) guyanensis* | 0.0239001315 | 0.0068705598 |
| *L. (V.) panamensis* | *L. (V.) guyanensis* | 0.0202242629 | 0.0061848609 |
| *L. (V.) shawi* | *L. (V.) guyanensis* | 0.0191201739 | 0.0061303404 |
| *L. (V.) braziliensis* | *L. (V.) naiffi* | 0.0368742747 | 0.0079827756 |
| *L. (V.) peruviana* | *L. (V.) naiffi* | 0.0359837654 | 0.0081746772 |
| *L. (V.) panamensis* | *L. (V.) naiffi* | 0.0420546151 | 0.0087096809 |
| *L. (V.) shawi* | *L. (V.) naiffi* | 0.0420505423 | 0.0088298466 |
| *L. (V.) guyanensis* | *L. (V.) naiffi* | 0.0465699035 | 0.0093349079 |
| *L. (V.) braziliensis* | *L. (V.) lainsoni* | 0.0469025997 | 0.0093586547 |
| *L. (V.) peruviana* | *L. (V.) lainsoni* | 0.0463838332 | 0.0095504119 |
| *L. (V.) panamensis* | *L. (V.) lainsoni* | 0.0449331240 | 0.0090631450 |
| *L. (V.) shawi* | *L. (V.) lainsoni* | 0.0539909210 | 0.0104981433 |
| *L. (V.) guyanensis* | *L. (V.) lainsoni* | 0.0523465324 | 0.0102919893 |
| *L. (V.) naiffi* | *L. (V.) lainsoni* | 0.0511004200 | 0.0099329331 |
| *L. (V.) braziliensis* | *L. (L.) mexicana* | 0.1739842329 | 0.0175430018 |
| *L. (V.) peruviana* | *L. (L.) mexicana* | 0.1731958763 | 0.0177527851 |
| *L. (V.) panamensis* | *L. (L.) mexicana* | 0.1682474227 | 0.0172523298 |
| *L. (V.) shawi* | *L. (L.) mexicana* | 0.1711340206 | 0.0174252783 |
| *L. (V.) guyanensis* | *L. (L.) mexicana* | 0.1777981313 | 0.0176754184 |
| *L. (V.) naiffi* | *L. (L.) mexicana* | 0.1690721649 | 0.0168949716 |
| *L. (V.) lainsoni* | *L. (L.) mexicana* | 0.1575257732 | 0.0165277758 |
| *L. (V.) braziliensis* | *L. (L.) venezuelensis* | 0.1656761674 | 0.0162087016 |
| *L. (V.) peruviana* | *L. (L.) venezuelensis* | 0.1659793814 | 0.0164076888 |
| *L. (V.) panamensis* | *L. (L.) venezuelensis* | 0.1610309278 | 0.0159346522 |
| *L. (V.) shawi* | *L. (L.) venezuelensis* | 0.1618556701 | 0.0160171979 |
| *L. (V.) guyanensis* | *L. (L.) venezuelensis* | 0.1674876696 | 0.0163288341 |
| *L. (V.) naiffi* | *L. (L.) venezuelensis* | 0.1635051546 | 0.0155366578 |
| *L. (V.) lainsoni* | *L. (L.) venezuelensis* | 0.1567010309 | 0.0156102661 |
| *L. (L.) mexicana* | *L. (L.) venezuelensis* | 0.0474226804 | 0.0077505705 |
| *L. (V.) braziliensis* | *L. (L.) amazonensis* | 0.1811356476 | 0.0162180022 |
| *L. (V.) peruviana* | *L. (L.) amazonensis* | 0.1819964798 | 0.0164149522 |
| *L. (V.) panamensis* | *L. (L.) amazonensis* | 0.1771653675 | 0.0159742498 |
| *L. (V.) shawi* | *L. (L.) amazonensis* | 0.1761629369 | 0.0159347067 |
| *L. (V.) guyanensis* | *L. (L.) amazonensis* | 0.1805027612 | 0.0162631583 |
| *L. (V.) naiffi* | *L. (L.) amazonensis* | 0.1797636409 | 0.0156262851 |
| *L. (V.) lainsoni* | *L. (L.) amazonensis* | 0.1786170480 | 0.0159412939 |
| *L. (L.) mexicana* | *L. (L.) amazonensis* | 0.0883580588 | 0.0108544167 |
| *L. (L.) venezuelensis* | *L. (L.) amazonensis* | 0.0547146090 | 0.0060857835 |
| *L. (V.) braziliensis* | *L. (L.) major* | 0.1680128144 | 0.0165504975 |
| *L. (V.) peruviana* | *L. (L.) major* | 0.1671434626 | 0.0167394633 |
| *L. (V.) panamensis* | *L. (L.) major* | 0.1730550549 | 0.0168424046 |
| *L. (V.) shawi* | *L. (L.) major* | 0.1637064903 | 0.0165640883 |
| *L. (V.) guyanensis* | *L. (L.) major* | 0.1760374882 | 0.0169742912 |
| *L. (V.) naiffi* | *L. (L.) major* | 0.1632940537 | 0.0160950197 |
| *L. (V.) lainsoni* | *L. (L.) major* | 0.1695114726 | 0.0165107208 |
| *L. (L.) mexicana* | *L. (L.) major* | 0.1552991526 | 0.0167064448 |
| *L. (L.) venezuelensis* | *L. (L.) major* | 0.1418154947 | 0.0149924870 |
| *L. (L.) amazonensis* | *L. (L.) major* | 0.1537164427 | 0.0151505396 |
| *L. (V.) braziliensis* | *L. (L.) donovani* | 0.1570219125 | 0.0165108607 |
| *L. (V.) peruviana* | *L. (L.) donovani* | 0.1562612825 | 0.0167546658 |
| *L. (V.) panamensis* | *L. (L.) donovani* | 0.1576055697 | 0.0168249044 |
| *L. (V.) shawi* | *L. (L.) donovani* | 0.1542794090 | 0.0167894133 |
| *L. (V.) guyanensis* | *L. (L.) donovani* | 0.1608807158 | 0.0169893360 |
| *L. (V.) naiffi* | *L. (L.) donovani* | 0.1551822205 | 0.0161754807 |
| *L. (V.) lainsoni* | *L. (L.) donovani* | 0.1492614446 | 0.0163975024 |
| *L. (L.) mexicana* | *L. (L.) donovani* | 0.1191538247 | 0.0151400527 |
| *L. (L.) venezuelensis* | *L. (L.) donovani* | 0.1129476820 | 0.0133907519 |
| *L. (L.) amazonensis* | *L. (L.) donovani* | 0.1334710552 | 0.0141192095 |
| *L. (L.) major* | *L. (L.) donovani* | 0.1081927262 | 0.0140476008 |
| *L. (V.) braziliensis* | *L. (L.) infantum* | 0.1613993197 | 0.0163159984 |
| *L. (V.) peruviana* | *L. (L.) infantum* | 0.1605462019 | 0.0165327557 |
| *L. (V.) panamensis* | *L. (L.) infantum* | 0.1619933492 | 0.0166305402 |
| *L. (V.) shawi* | *L. (L.) infantum* | 0.1586607264 | 0.0165049047 |
| *L. (V.) guyanensis* | *L. (L.) infantum* | 0.1651680992 | 0.0166977180 |
| *L. (V.) naiffi* | *L. (L.) infantum* | 0.1549581943 | 0.0160257504 |
| *L. (V.) lainsoni* | *L. (L.) infantum* | 0.1486959882 | 0.0162320983 |
| *L. (L.) mexicana* | *L. (L.) infantum* | 0.1196917321 | 0.0150277354 |
| *L. (L.) venezuelensis* | *L. (L.) infantum* | 0.1165984237 | 0.0133567943 |
| *L. (L.) amazonensis* | *L. (L.) infantum* | 0.1402160838 | 0.0142337027 |
| *L. (L.) major* | *L. (L.) infantum* | 0.1149528055 | 0.0143706850 |
| *L. (L.) donovani* | *L. (L.) infantum* | 0.0145825414 | 0.0055332804 |
| *L. (V.) braziliensis* | *L. (L.) tropica* | 0.1697392359 | 0.0168231105 |
| *L. (V.) peruviana* | *L. (L.) tropica* | 0.1690721649 | 0.0170559425 |
| *L. (V.) panamensis* | *L. (L.) tropica* | 0.1782817869 | 0.0174735336 |
| *L. (V.) shawi* | *L. (L.) tropica* | 0.1711340206 | 0.0172146059 |
| *L. (V.) guyanensis* | *L. (L.) tropica* | 0.1779126788 | 0.0172030195 |
| *L. (V.) naiffi* | *L. (L.) tropica* | 0.1748453608 | 0.0167793293 |
| *L. (V.) lainsoni* | *L. (L.) tropica* | 0.1703092784 | 0.0165049491 |
| *L. (L.) mexicana* | *L. (L.) tropica* | 0.1340206186 | 0.0157810587 |
| *L. (L.) venezuelensis* | *L. (L.) tropica* | 0.1340206186 | 0.0148271875 |
| *L. (L.) amazonensis* | *L. (L.) tropica* | 0.1535328137 | 0.0152562952 |
| *L. (L.) major* | *L. (L.) tropica* | 0.1181798520 | 0.0148737893 |
| *L. (L.) donovani* | *L. (L.) tropica* | 0.0757108258 | 0.0121394253 |
| *L. (L.) infantum* | *L. (L.) tropica* | 0.0805098257 | 0.0125062533 |
| *L. (V.) braziliensis* | *L. colombiensis* | 0.1718369439 | 0.0173382389 |
| *L. (V.) peruviana* | *L. colombiensis* | 0.1711340206 | 0.0175214830 |
| *L. (V.) panamensis* | *L. colombiensis* | 0.1744740889 | 0.0175471194 |
| *L. (V.) shawi* | *L. colombiensis* | 0.1732299565 | 0.0176411913 |
| *L. (V.) guyanensis* | *L. colombiensis* | 0.1736768880 | 0.0175222883 |
| *L. (V.) naiffi* | *L. colombiensis* | 0.1665683613 | 0.0164693787 |
| *L. (V.) lainsoni* | *L. colombiensis* | 0.1620713495 | 0.0167520449 |
| *L. (L.) mexicana* | *L. colombiensis* | 0.2000590319 | 0.0179449592 |
| *L. (L.) venezuelensis* | *L. colombiensis* | 0.1845905500 | 0.0168796549 |
| *L. (L.) amazonensis* | *L. colombiensis* | 0.1952151821 | 0.0169275277 |
| *L. (L.) major* | *L. colombiensis* | 0.1793042646 | 0.0169055159 |
| *L. (L.) donovani* | *L. colombiensis* | 0.1717131312 | 0.0167551761 |
| *L. (L.) infantum* | *L. colombiensis* | 0.1773953484 | 0.0167546067 |
| *L. (L.) tropica* | *L. colombiensis* | 0.1938716391 | 0.0178543516 |
| *L. (V.) braziliensis* | *L. equatorensis* | 0.1718010916 | 0.0173327159 |
| *L. (V.) peruviana* | *L. equatorensis* | 0.1711340206 | 0.0175193357 |
| *L. (V.) panamensis* | *L. equatorensis* | 0.1744329897 | 0.0175413108 |
| *L. (V.) shawi* | *L. equatorensis* | 0.1731958763 | 0.0176352457 |
| *L. (V.) guyanensis* | *L. equatorensis* | 0.1736739466 | 0.0175198950 |
| *L. (V.) naiffi* | *L. equatorensis* | 0.1665979381 | 0.0164673700 |
| *L. (V.) lainsoni* | *L. equatorensis* | 0.1620618557 | 0.0167490728 |
| *L. (L.) mexicana* | *L. equatorensis* | 0.2000000000 | 0.0179382106 |
| *L. (L.) venezuelensis* | *L. equatorensis* | 0.1845360825 | 0.0168729630 |
| *L. (L.) amazonensis* | *L. equatorensis* | 0.1953231079 | 0.0169326124 |
| *L. (L.) major* | *L. equatorensis* | 0.1792513485 | 0.0169004809 |
| *L. (L.) donovani* | *L. equatorensis* | 0.1717032814 | 0.0167545946 |
| *L. (L.) infantum* | *L. equatorensis* | 0.1773917600 | 0.0167546229 |
| *L. (L.) tropica* | *L. equatorensis* | 0.1938144330 | 0.0178484499 |
| *L. colombiensis* | *L. equatorensis* | 0.0000000000 | 0.0000000000 |
| *L. (V.) braziliensis* | *L. hertigi* | 0.1675560946 | 0.0167971582 |
| *L. (V.) peruviana* | *L. hertigi* | 0.1690721649 | 0.0170022468 |
| *L. (V.) panamensis* | *L. hertigi* | 0.1703092784 | 0.0169413307 |
| *L. (V.) shawi* | *L. hertigi* | 0.1587628866 | 0.0166518486 |
| *L. (V.) guyanensis* | *L. hertigi* | 0.1673731220 | 0.0169190593 |
| *L. (V.) naiffi* | *L. hertigi* | 0.1616494845 | 0.0160870459 |
| *L. (V.) lainsoni* | *L. hertigi* | 0.1632989691 | 0.0167877972 |
| *L. (L.) mexicana* | *L. hertigi* | 0.1917525773 | 0.0176518521 |
| *L. (L.) venezuelensis* | *L. hertigi* | 0.1783505155 | 0.0164528711 |
| *L. (L.) amazonensis* | *L. hertigi* | 0.1904450591 | 0.0165128416 |
| *L. (L.) major* | *L. hertigi* | 0.1901973378 | 0.0173035169 |
| *L. (L.) donovani* | *L. hertigi* | 0.1729403948 | 0.0167037378 |
| *L. (L.) infantum* | *L. hertigi* | 0.1671224608 | 0.0161970034 |
| *L. (L.) tropica* | *L. hertigi* | 0.1896907216 | 0.0175081038 |
| *L. colombiensis* | *L. hertigi* | 0.1196229263 | 0.0145943792 |
| *L. equatorensis* | *L. hertigi* | 0.1195876289 | 0.0145894151 |
