# Supplementary material for: Applying a cytochrome c oxidase I barcode for Leishmania species typing
Source: PLoS One. 2024 Dec 2;19(12):e0309277. doi: 10.1371/journal.pone.0309277 (PMC11611135; doi:10.1371/journal.pone.0309277)
Supplement: Attachment — Submitted filename: Response _to_reviewers_PlosOne_2024.doc [file pone.0309277.s003.doc]

**Response to Reviewers**

September 24^th^, 2024

Dear Editors of Plos One,

We wish to submit the corresponding response to the questions and comments stated by the reviewers to our research article entitled “Applying a cytochrome oxidase I barcode for *Leishmania* species typing” for consideration by Plos One:

Reviewer #1: In this manuscript, the authors describe the use of the mitochondrial gene Cytochrome Oxidase I for Leishmania typing. The manuscript is clear and well-written. I have some suggestions that I consider that may improve the quality of this study.

Main points:

1) It is not clear in the manuscript whether the primers designed for amplification of conserved region of coI are able to amplify coI gene of Trypanosomatidae family members, as Trypanosoma cruzi, T. brucei. Could you clarify this point in the manuscript. Are these primers specific for Leishmania genus?

Response: The primers were designed to amplify the family Trypanosomatidae, they are not specific to *Leishmania*. We avoided designing a genus-specific primer in order to generate lines of research, in the near future, involving different members of the family. For example, one paper of some colleagues of ours has already been published, applying the same primers to *T. cruzi* with a few modifications. We will clarify this situation in the manuscript, so there’s no confusion. We hope to keep working with this *Leishmania* research line in order to improve and optimize the proposed PCR protocol.

2) In Table 2, it is indicated that the amplicon size is 486 bp, while in Results, the amplicon has around 600bp. Please, include the expected size of this amplicon in Table 2. Are there differences in size of this amplicon among Leishmania species? If not, what are the variations in size? Could you include this information in the manuscript?

Response: We took the opportunity given by Reviewer #1’ last recommendation and propose a schematic map of the gene and its corresponding multiple alignment. In this figure we thoroughly explain the characteristics of the amplicon for which we have a calculated size of 572 bp for all related trypanosomatids analyzed. After processing, eliminating the ends and clearing low quality regions, *Leishmania* species consistently show a 486 bp amplicon, suitable for genetic sequencing.

3) I would also suggest to include a figure in the manuscript containing the ORF of coI gene and the location of these primers.

Response: Following this recommendation, we included a new figure in the manuscript with all corresponding information regarding *coI* gene.

Reviewer #2: In the article “Applying a cytochrome c oxidase I barcode for Leishmania species typing” the authors study the COI gene as a molecular marker for phylogenetic analysis and characterization of Leishmania species in order to build a genetically supported taxonomy for the entire genus and obtain a marker for Leishmania species identification. Therefore, it is necessary to agree on a practical taxonomic classification scheme based on reliable and consensual concepts. A good definition of Leishmania species is crucial for the correct diagnosis and prognosis of the disease, as well as for making decisions about treatment and control measures. The article contribute to the taxonomic discussion of Leishmania genus and provide and other potential gene for Leishmania species identification. The useful of this marker for the detection and identification directly for clinical samples will be study in a future. The article is very well writing and the results obtained are very well discussed.

Response: We are thankful to Reviewer #2 for his kind words regarding this article, an effort that took years in the making, and that we finally get to published.

With the changes implemented in the manuscript we believe that this article is appropriate for publication by Plos One.

We thank you and the reviewers for the time and effort spent in reviewing this document. We consider the feedback as invaluable in improving the content and presentation of the paper.

Thank you for your consideration of this manuscript.

Sincerely,

Carlos Alberto Mata Somarribas
Instituto Costarricense de Investigación y Enseñanza en Nutrición y Salud
100m oeste del Servicentro Tinoco, Cartago, Costa Rica
